# Comparison of Biological, Pharmacological Characteristics, Indications, Contraindications, Efficacy, and Adverse Effects of Inactivated Whole-Virus COVID-19 Vaccines Sinopharm, CoronaVac, and Covaxin: An Observational Study

**DOI:** 10.3390/vaccines11040826

**Published:** 2023-04-11

**Authors:** Sultan Ayoub Meo, Riham A. ElToukhy, Anusha Sultan Meo, David C. Klonoff

**Affiliations:** 1 Department of Physiology, College of Medicine, King Saud University, Riyadh 11461, Saudi Arabia; 2 Department of Family Medicine, College of Medicine, King Saud University, Riyadh 2925, Saudi Arabia; 3 College of Medicine, King Saud University, Riyadh 11461, Saudi Arabia; 4 Diabetes Research Institute, Mills-Peninsula Medical Center, San Mateo, CA 94010, USA

**Keywords:** SARS-CoV-2, vaccines, pharmacology, adverse effects

## Abstract

Severe acute respiratory syndrome coronavirus 2 (SARS-CoV-2) is an emerging viral zoonotic illness that has developed a distinctive and threatening situation globally. Worldwide, many vaccines were introduced to fight against the COVID-19 pandemic. The present study aims to compare the bio-pharmacological characteristics, indications, contraindications, efficacy, and adverse effects of inactivated whole-virus COVID-19 vaccines, Sinopharm, CoronaVac, and Covaxin. Initially, 262 documents and 6 international organizations were selected. Finally, 41 articles, fact sheets, and international organizations were included. The data were recorded from the World Health Organization (WHO), Food and Drug Administration (FDA) USA, Web of Science, PubMed, EMBASE, and Scopus. The results demonstrated that these three inactivated whole-virus COVID-19 vaccines, Sinopharm, CoronaVac, and Covaxin, received emergency approval from the FDA/WHO, and all three of these vaccines are beneficial for the prevention of the COVID-19 pandemic. The Sinopharm vaccine has been recommended during pregnancy and for people of all age groups, and the CoronaVac and Covaxin vaccines are recommended for people over 18 years of age and older. These three vaccines have recommended intramuscular doses of 0.5 mL each, with a 3–4 week interval. These three vaccines can be stored in a refrigerator at +2 to +8 °C. The common adverse effects of these vaccines are pain at the injection site, redness, fatigue, headache, myalgias, general lethargy, body ache, arthralgia, nausea, chills, fever, and dizziness. The overall mean efficiency for the prevention of the COVID-19 disease is 73.78% for Sinopharm, 70.96% for CoronaVac, and 61.80% for Covaxin. In conclusion, all three inactivated whole-virus COVID-19 vaccines, Sinopharm, CoronaVac, and Covaxin, are beneficial for the prevention of the COVID-19 pandemic. However, evidence suggests that the overall impact of Sinopharm is slightly better than that of CoronaVac and Covaxin.

## 1. Introduction

Severe acute respiratory syndrome coronavirus 2 (SARS-CoV-2) has developed a highly threatening situation worldwide. The swift spread of the disease is due to its numerous epidemiological and transmission trends. A SARS-CoV-2 infection is extremely contagious and provokes extensive health and socio-economic harm [1]. From 19 December 2019 to 25 March 2023, the virus swiftly spread all around the world and infected 761,402,282 people, resulting in 6,887,000 deaths with a fatality rate of 0.90% [2].

The global epidemiological and transmission trends of the disease are linked to various factors, including social gathering, travel, close contact, body fluids, respiratory droplets, contaminated objects, seasonal variations, contagious environment, and environmental pollution [3,4]. Currently, the SARS-CoV-2 disease has caused multiple challenges for healthcare officials because there is no specific line of treatment. Vaccination and potential herd immunity also in some settings is the most effective strategy against the COVID-19 pandemic and protects people from this global pandemic [5].

The COVID-19 vaccinations were developed in an abbreviated time, and the worldwide public expressed some apprehensions about the interim authorization of COVID-19 vaccines. Limited literature in referenced medical journals on PubMed has evaluated the efficiency of vaccines in the prevention of the pandemic. However, the risk–benefit balance of the COVID-19 vaccination still needs further evidence-based clarification [6].

The US Food and Drug Administration (FDA) has given interim authorization for the various types of vaccines including mRNA, vector, inactivated, and subunit-type vaccines against the COVID-19 pandemic [7]. Worldwide, 5.51 billion people received at least a single dose of a COVID-19 vaccine, equal to 81.8% of the world population. About 13.25 billion doses of vaccines have been administered globally, and 1.44 million people per day are now being vaccinated against COVID-19. Nevertheless, in low-income developing nations, the vaccination rate is still very low, and about 26.4% of people in those countries have received at least one dose [8,9]. The literature is insufficient to establish a comprehensive comparison between the various types of vaccines. The inactivated whole-virus COVID-19 vaccine group contains three vaccines, which are commonly used in many countries. Therefore, this study aims to compare the biological and pharmacological characteristics, indications, contraindications, efficacy, and adverse effects of three different inactivated whole-virus COVID-19 vaccines: Sinopharm, CoronaVac, and Covaxin.

## 2. Materials and Methods

This study was organized within the Department of Physiology, College of Medicine, King Saud University, Riyadh, Saudi Arabia. This study focused on three inactivated vaccines. Inactivated vaccines are commonly used in many countries worldwide. Sinopharm, Sinovac CoronaVac, and Covaxin were reviewed.

In this study, the data were obtained from wide-reaching, very trustworthy, and evidence-based organizations and websites, which highlight the bio-pharmacological characteristics, indications, contraindications, efficacy, and side effects of three inactivated whole-virus COVID-19 vaccines, namely the Sinopharm, CoronaVac, and Covaxin vaccines. The data were recorded from the World Health Organization-WHO, US Food and Drug Authorities-FDA, Fact Sheets, Clarivate Analytics Web of Science, Medline, EMBASE, PubMed, and Scopus. The data were filtered by using the key terms SARS-CoV-2, COVID-19 vaccines, Sinopharm, CoronaVac, and Covaxin vaccines, pharmacological characteristics, indications, contraindications, efficacy, immunogenicity, and adverse events.

**Figure 1:**
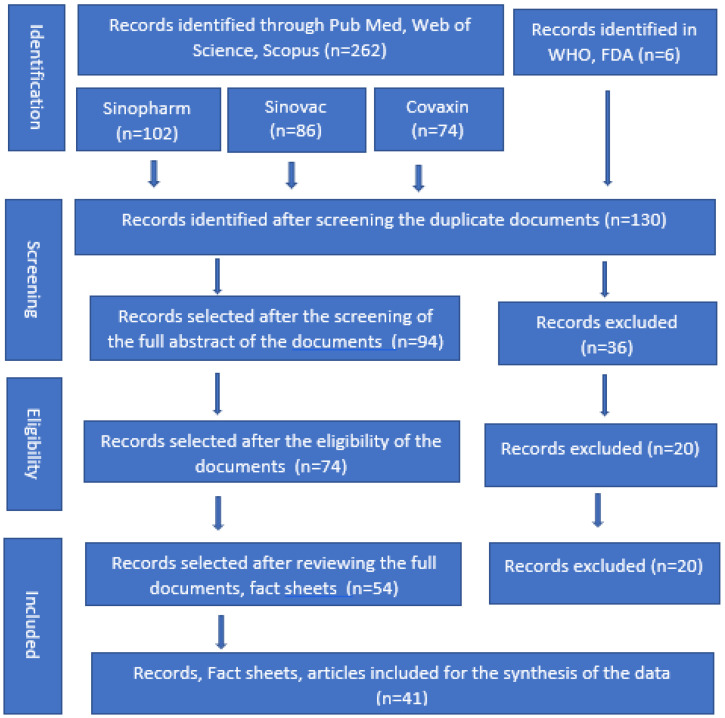
PRISMA flow diagram for the selection of fact sheets and documents about Sinopharm, CoronaVac, and Covaxin COVID-19 vaccines.

In this study, initially, 262 documents and 6 international organizations (a total of 268) were selected, and documents were included from publicly available web databases, including PubMed, Web of Science, Scopus, and Google Scholar. After reviewing the abstracts and summary reports, 82 documents were selected for detailed review and finally, 41 articles, fact sheets, and international organizations’ websites, including the websites of the World Health Organization (WHO) and the US Food and Drug Administration (FDA) were selected for the data analysis (the study-filtering process is illustrated in Figure 1). There were no limitations on the study design, type, or publication language. The essential pieces of evidence and data were recorded from the selected organizations and documents. One investigator appraised the articles, recorded the information, and entered the findings into tabular form. After that, another team member rechecked the findings.

Ethics statement: The findings were documented from information publicly available from selected government and non-profit organizations, and other databases on the topics of the Sinopharm, CoronaVac, and Covaxin COVID-19 vaccines. Hence, ethical approval was not required.

## 3. Results

Table 1 shows the biological and pharmacological characteristics of the inactivated whole-virus COVID-19 vaccines Sinopharm, CoronaVac, and Covaxin. The FDA has provided emergency authorization for the administration of the Sinopharm, CoronaVac, and Covaxin COVID-19 vaccines. FDA approval was granted for Sinopharm on 7 May 2021, updated 14 October 2021; 15 March 2022; CoronaVac/Sinovac was approved on 24 May 2021; and Covaxin on 3 November 2021. The dose of all of these vaccines is two doses (0.5 mL each) intramuscularly with an interval of 3–4 weeks and a booster dose 4–6 months following the primary series vaccination. The approximate cost of Sinopharm was about USD 30 (GBP 22–26) per dose, the cost of CoronaVac was USD 5–14 per dose, and the cost of the Covaxin COVID-19 vaccine was about USD 2 per dose (Table 1).

The major local complaints of Sinopharm may include pain or redness at the injection site, fatigue, headache, myalgias, general lethargy, body ache, arthralgia, nausea, chills, fever, and dizziness (Table 2).

The common adverse effects of the CoronaVac/Sinovac vaccine can include injection site pain, fatigue, headache, muscle pain, and joint pain. Moreover, it can cause thromboembolism (Table 2). Finally, the adverse effects of the Covaxin vaccine can include local injection site pain, swelling, redness, and itching. The systemic effects can include headache, fever, malaise, body aches, nausea, and vomiting. The overall adverse effects were mild or moderate and common after the first dose. However, a severe allergic reaction may very rarely occur after the first dose of the COVAXIN vaccine (Table 2).

**Figure 2:**
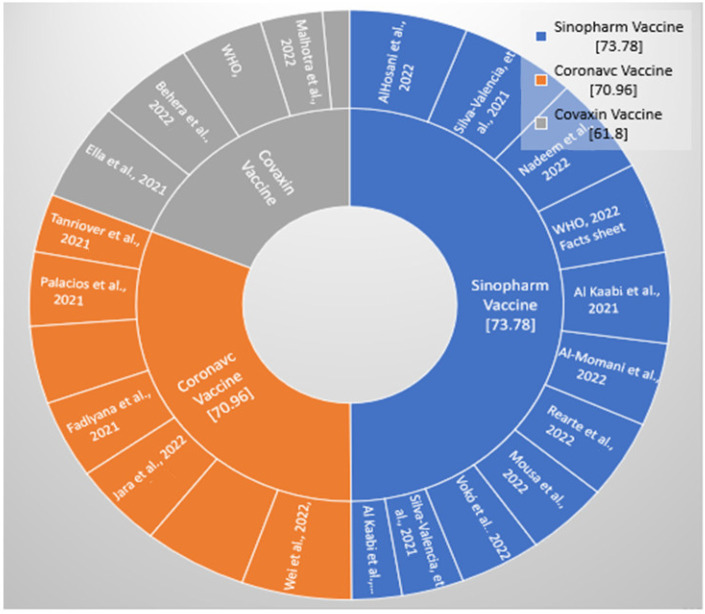
Distribution of studies selected for the efficacy of the Sinopharm, CoronaVac, and Covaxin vaccines for the prevention of COVID-19 [10,11,14,15,29,30,31,32,34,35,36,37,40,41,42,43,44,47,48,49].

**Figure 3:**
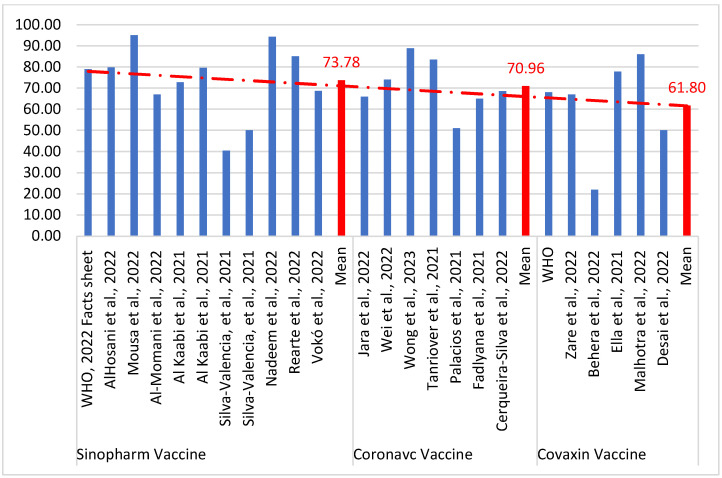
Efficacy of the Sinopharm, CoronaVac, and Covaxin vaccines for the prevention of COVID-19 [29,30,31,32,33,34,35,36,37,38,39,40,41,42,43,44,45,46,47,48,49,50].

Table 3 demonstrates the efficacy of the three different inactivated whole-virus COVID-19 vaccines, Sinopharm, CoronaVac, and Covaxin. Based on the available data, it was determined that after the two doses of Sinopharm, with an interval of 21 days, efficacy was 73.78%, and it was effective against new virus variants and for persons aged 60 years and above with comorbidities. Two doses of CoronaVac/Sinovac at an interval of 14 days (2–4 weeks) has an efficacy of 70.96 against SARS-CoV-2 symptomatic patients. Moreover, the mean efficacy of Covaxin is 61.80% against all variants of COVID-19 (Table 3; Figure 2 and Figure 3).

**Table 3:** Efficacy of Sinopharm, CoronaVac, and Covaxin COVID-19 vaccines in the prevention of COVID-19 cases and hospital admission.

Author Name, Year	Country, Type of Study	Age Range	Efficacy
Sinopharm Vaccine			
WHO, 2022 [10] Facts sheet [11]	China, RCT phase 3	18–59 years	Efficacy after 2 doses with 14–21-day interval was 79% (CI: 66–87%)
AlHosani et al., 2022 [29]	UAE, cohort study	15 years and above	Prevent hospitalization = 79.8% (78~81.4%); critical care = 92.2% (89.7~94.1%; deaths = 97.1% (83~99.9%)
Mousa et al., 2022 [30]	UAE, evidence-based	3782, above 18 years	Full vaccination prevents hospital admission against Delta variant = 95% (94–97%)
Al-Momani et al., 2022 [31]	Jordan, cross-sectional	536, over 18 years	Sinopharm vaccine efficacy was 67% (95% CI 52–78%)
Al Kaabi et al., 2021 [32]	Asia, phase 3 trial	40,382 participants	72.8% (95% CI, 58.1–82.4%) for WIV04 and 78.1% (95% CI, 64.8–86.3%) for HB02 symptomatic COVID-19 cases
Al Kaabi et al., 2021 [33]	UAE, randomized phase 3 trial	3,147,869 adults >18 years	Effectiveness was 79.6% (CI: 77.7–81.3) against hospitalization, 86% (CI: 82.2–89.0) against critical care admission, and 84.1% (CI: 70.8–91.3) against death due to COVID-19
Silva-Valencia et al., 2021 [34]	Peru, retrospective		Infection 40.3% (38.9–41.6%); COVID-19 mortality 88.7% (85.1–91.4%)
Silva-Valencia et al., 2021 [35]	Peru, cohort study		Effectiveness was 50% (CI: 49–52%) against infection and 94% (95% CI: 91–96%) against COVID-19-allied mortality
Nadeem et al., 2022 [36]	Case-control, Pakistan	3426, aged >60 years	Efficacy against symptomatic COVID-19 infection was 94.3%
Rearte et al., 2022 [37]	Retrospective, Argentina	237,330, >60 years old	Efficacy against symptomatic COVID-19 infection was 85.0% (84.0–86.0)
CoronaVac/Sinovac Vaccine			
Wei et al., 2022 [38]	Case-control, Hong Kong	32,823 cases ≥65 years	Hospitalization 74.0% (95% CI, 71.8–75.8%). Deaths 86.4% (95% CI, 85.8–87.0%)
Wong et al., 2023 [39]	National data, Malaysia	1,158,235 >18 years	CoronaVac is 88.8% (CI 95%: 84.9, 91.7)
Jara et al., 2022 [40]	Chile, Cohort ≥18 years	10.2 million	Overall efficacy was 65.9% (65.2–66.6); for prevention of hospitalization, it was 87.5% (86.7–88.2); for ICU admission, it was 90.3% (CI: 89.1–91.4); for COVID deaths, it was 86.3% (CI: 84.5–87.9)
Tanriover et al., 2021, [41]	Turkey, RCT	13,000 RCT ≥18 years	Protection against symptomatic disease 83.5% (65–92); 100% (65–92); hospitalization 100% (20–100)
Fadlyana et al., 2021 [42]	Indonesia, RCT	1620 ≥18 years	Protection against symptomatic disease 65% (20–85)
Palacios et al., 2021 [43]	Brazil, RCT	12,688 ≥ 18 years	Protection against symptomatic disease 51% (36–62); protection against hospitalization 100% (56–100)
Vokó et al., 2022 [44]	Hungry, retrospective	895,465	Estimated effectiveness against SARS-CoV-2 infection was 68.7% (95% CI 67.2%-70.1%)
Cerqueira-Silva et al., 2022 [45]	Brazil, case control	14,362,482	Efficacy at 14–30 days after the second dose was 55.0% (CI: 54.3–55.7) against confirmed infection and 82.1% (95% CI: 81.4–82.8) against severe outcomes (mean 68.55)
Covaxin Vaccine			
WHO [14,15]	-	-	68%; all variants of COVID-19 were 71% (CI: 50–84); Kappa 90% (95% CI: 30–100); and Delta 65% (95% CI: 33–83).
Zare et al., 2022 [46]	Iran	214 people 19–64 years	67%
Behera et al., 2022 [47]	Case-control study, India	670 people, 29.1 years	After age and gender adjustment, vaccine effectiveness was 22% CI: 0.52–1.17; 29% (CI: 0.47–1.08)
Ella et al., 2021 [48]	Phase 3 clinical trial	25,798, age ≥ 18 years	Overall vaccine efficacy was 77·8% (95% CI 65·2–86·4).
Malhotra et al., 2022 [49]	Retrospective cohort, India	15,244 HCWs, age 36.6 years	The efficacy against the infection was 86% (95% CI, 77–92%)
Desai et al., 2022 [50]	Case-control study, India	3732 > 18 years	Adjusted efficacy against symptomatic cases after 2 doses was 50% (95% CI 33–62)

## 4. Discussion

Severe acute respiratory syndrome coronavirus 2 (SARS-CoV-2) has developed into a highly distinctive, challenging, and threatening situation. Vaccines are the best strategy to protect people from this pandemic. Worldwide, people have received vaccinations, but there is a need to gather evidence about the safety and adverse effects of these vaccines. The immunity induced by these vaccines not only depends on the host factors but also is determined by vaccine components. Therefore, it is vital to understand the biological and pharmacological characteristics, efficacy, and adverse effects of various vaccines. We studied all three vaccines of this inactivated group (Sinopharm, CoronaVac, and Covaxin) because the three vaccines are used in many countries by people with diverse socio-economical, genetic, and environmental conditions.

The Sinopharm vaccine was prepared in China and was added to the WHO emergency use list. The Sinopharm vaccine has been widely distributed, and some clinical trials were conducted in various regions worldwide. The clinical trials in children showed this vaccine to be safe and demonstrated robust humoral responses against the SARS-CoV-2 infection after two doses of the vaccine [51].

AlHosani et al., 2022 [29] investigated the efficacy of the Sinopharm vaccine among people in Abu Dhabi, UAE. The study population was people 14 days post-vaccination who were either fully or partially vaccinated. The efficacy of the Sinopharm vaccine among fully vaccinated people was 80% against hospitalization, 92% against severe diseases, and 97% against death [29]. In another study also conducted in the United Arab Emirates, the Sinopharm vaccine demonstrated disease prevention and hospitalizations due to the SARS-CoV-2 Delta variant with an effectiveness of about 95% (95% CI, 94% to 97%) [30].

These studies of the efficacy of Sinopharm vaccines demonstrate satisfactory effectiveness. For further understanding of the effectiveness of the Sinopharm vaccine, clinical trials were organized during the first wave of the pandemic and demonstrated that the Sinopharm vaccine offered satisfactory effectiveness in preventing SARS-CoV-2 cases and deaths. The estimated disease efficacy was 78.89% (95% CI, 65.79%–86.97%). Moreover, the vaccine efficacy estimates were similar in males at 78.4% and females at 75.6% [52]. A study conducted in Peru reported a similar efficacy in preventing both cases and COVID-19-specified mortality. These protections were, respectively, 90.5% and 93.9% [35].

Phase three clinical trials in the literature from various countries have shown that the two doses, with a 21-day interval, have an efficacy of 79% (CI: 66–87%) against SARS-CoV-2 symptomatic cases and an efficacy of 79% (95% CI: 26–94%) against hospitalization [32]. Jara et al., 2022 [40] conducted a cohort study on the efficacy of CoronaVac vaccines and recruited about 10.2 million persons. The adjusted vaccine effectiveness was 65.9% (65.2 to 66.6) against COVID-19 disease; 87.5% (86.7 to 88.2) against hospitalization; 90.3% (CI, 89.1–91.4) against ICU admission; and 86.3% (CI: 84.5–87.9) for preventing COVID-deaths.

Meo et al., 2023 [20] studied the adverse effects of the first and second doses of the Sinopharm vaccine among vaccinated medical students and healthcare workers. The common side effects were pain at the injection site, general lethargy, myalgia/body pain, low-grade fever, and headache. The adverse effects were mild in intensity for both doses but slightly more common and severe after the first dose than the second dose of the vaccine [20].

In another study conducted on Sinopharm in Iran, Almufty et al., 2021 [53] found that with both doses of vaccines, females reported more adverse effects compared to males. These adverse effects were similar to those identified in a study conducted in the United Arab Emirates [54]. The most frequently reported adverse effects were fatigue, chill, fever, headache, and injection site reactions.

Babaee et al., 2022 [55] performed a study in Iran among persons who received the Sinopharm vaccine. Among the 979 participants, 62.6% of the Sinopharm recipients did not report adverse effects after the first or second doses. The commonly reported adverse effects of the Sinopharm vaccine were fatigue, chill, fever, dizziness, headache, and local reactions.

The other vaccine, Sinovac, commonly called CoronaVac, has also been widely used against the COVID-19 pandemic. The overall efficacy for the prevention of symptomatic cases was 67.7% (95% CI, 35.9% to 83.7%). Studies in three countries reported the effectiveness of the prevention of symptomatic cases after the second dose. The effectiveness in Turkey was 83.5% (95% CI, 65.4% to 92.1%) [41], in Brazil, it was 50.7% (95% CI, 35.9–62.0%) [43], and in Indonesia, it was 65% (95% CI, 20% to 85%) [42,56]. The overall efficacy for the prevention of symptomatic COVID-19 was 67.7% (95% CI, 35.9% to 83.7%). Moreover, a study conducted in Chile reported that the vaccine efficacy against COVID-19 infection was 66.6%, against hospitalization, it was 85.3%, and against ICU admission, it was 89.2% [57].

The Covaxin (BBV152) vaccine is also a whole-virion-inactivated SARS-CoV-2 vaccine against the COVID-19 pandemic. It enhances the human body’s immune system without the risk of causing the disease. The mechanism of action is based on the principle that once inactivated viruses enter the body, they enhance antibody production and ensure that the body is ready to respond to an infection with live SARS-CoV-2. This vaccine contains an aluminum-based adjuvant to enhance the immune system’s response [British Society of Immunology] [58].

A study by Ella et al., 2021 [48] reported the Covaxin vaccine’s efficacy among Indian people who were vaccinated two weeks after their second vaccination to be 77.8%. In another study, Singh et al. [18] investigated the antibody response after the Covaxin vaccine among healthcare workers in India. The authors reported that participants showed seropositivity after vaccination, which was almost 70%. Similarly, Kumar et al., 2021 [59] completed a study on the antibody responses to the BBV152 vaccine among healthcare professionals. The investigators found that about 76% of participants showed seropositivity after vaccination [59].

The literature demonstrates that the efficacy of the Covaxin vaccine was 63% in asymptomatic people, 78% for mild, moderate, and severe cases, 65% for the Delta variant, and 93% for severe COVID-19 cases. The Covaxin vaccine was also found to be effective in neutralizing this Alpha strain, and the vaccine was found to have a neutralizing capacity regarding the Beta and Delta variants [60]

## 5. Study Strengths and Limitations

This study establishes a comparison between various characteristics, efficacies, and adverse effects of three inactivated whole-virus COVID-19 vaccines, Sinopharm, CoronaVac, and Covaxin. This article provides a detailed comparison for a better understanding of these three vaccines in the prevention of COVID-19 disease. Moreover, the analysis of the findings is based on reputable evidence-based documents. The insights of this study are important for policymakers. The limitations of the present study include a limited amount of evidence about, first, the efficacy and adverse effects of these vaccines, and second, the specific virus variants-based information that could not be determined. Third, little is known about the vaccine in regions with low-income and middle-income countries, and this information might be missed despite a search for data from every country about responses to these vaccines. The above limitations must be considered when interpreting our reported vaccine outcomes.

## 6. Conclusions

The FDA has granted emergency use of the Sinopharm, CoronaVac, and Covaxin COVID-19 vaccines in adults 18 years of age and older. All these vaccines are inactivated vaccines and contain the killed virus. The Sinopharm, CoronaVac, and Covaxin COVID-19 vaccines are effective in reducing rates of infection and hospitalization and are thus beneficial for the prevention of the COVID-19 pandemic. The common adverse effects of these three vaccines are pain and redness at the injection site, fatigue, headache, myalgia, general lethargy, body ache, arthralgia, nausea, chills, fever, and dizziness. Furthermore, the CoronaVac/Sinovac vaccine may also cause thromboembolism. The evidence supports the conclusion that the overall benefit of Sinopharm is slightly greater than that of CoronaVac and Covaxin. This research emphasizes the importance of continuously monitoring vaccine efficacy and adverse events. This study encourages future studies on Sinopharm, CoronaVac, and Covaxin vaccines and will lead to making a comparison between different vaccines to establish a better understanding of the vaccination outcomes.

## Figures and Tables

**Table 1:** Comparison of biological and pharmacological characteristics, indications, contraindications, and mechanisms of action of the COVID-19 vaccines Sinopharm, CoronaVac, and Covaxin.

Characteristics	Sinopharm	CoronaVac/Sinovac	Covaxin
Generic name	Sinopharm [10,11]	CoronaVac [12,13]	Covaxin vaccine [14,15]
Brand name	BIBP-CorV [10,11]	COVID-19 Vero Cell [12,13]	BBV152, COVAXIN [14,15]
Type of Vaccine	Whole-virion inactivated vaccine [10,11]	Whole-virion inactivated vaccine [12,13]	Whole-virion inactivated vaccine [14,15]
Manufacturer, country	CNPGC, Beijing, China [10,11]	Sinovac Biotech, China [12,13]	Bharat Biotech, India [14,15]
FDA/WHO approval	7 May 2021, updated 14 October 2021; 15 March 2022 [10,11]	24 May 2021 [12,13]	3 November 2021 [14,15]
Dose(s)	Two doses, 0.5 mL each, with a 3–4 week interval [10,11]	Two doses, 0.5 mL each, with a 28-day interval [12,13]	Two doses, 0.5 mL each, with a 28-day interval [14,15]
Booster shots	4–6 months following primary series vaccination [10,11]	4–6 months following primary series vaccination [12,13]	4–6 months following primary series vaccination [14,15]
Route of administration	Intramuscular injection [10,11]	Intramuscular injection [12,13]	Intramuscular injection [14,15]
Storage	Store the box in a refrigerator at +2 to +8 °C. [10,11]	Store the box in a refrigerator at +2 to +8 °C [12,13]	Store the box in a refrigerator at +2 to +8 °C [14,15]
Vaccination cost	About USD 30 (GBP 22–26) per dose [10,11]	About USD 5–14 per dose [12,13]	About USD 2 per dose [14,15]
Effectiveness	Two doses at an interval of 21 days, efficacy 79% (CI: 66–87%); 84% (CI: 80–88%); 86% (CI: 80–91%); and 94% (CI: 62–100%) [10,11]	In two doses, with 2–4 week intervals, efficacy was 51% (CI: 36–62%) in symptomatic patients; 100% (CI: 17–100%) in severe cases; and 100% (CI: 56–100%) against hospitalization [12,13]	68% for all variants of COVID-19 and 71% (CI: 50–84%); Kappa 90% (95% CI: 30–100%); and Delta 65% (95% CI: 33–83%) [14,15]
Effective age	18 years and above [10,11]	18 years and above [12,13]	18 years and above [14,15]
Pregnant females	WHO suggests the use of the vaccine in pregnant women when the benefits outweigh the potential risks [10,11]	Pregnant women data are lacking. WHO suggests use in pregnancy, when the benefits outweigh the risks [12,13]	Data on pregnant women are insufficient. Minor adverse events were found [14,15]
Breastfeeding	WHO suggests use in breastfeeding women [10,11]	WHO recommends the use in lactating women as in other adults [12,13]	WHO recommends use in lactating women as in other adults [14,15]
People with comorbidities	Data are insufficient [10,11]	Recommended for persons with comorbidities [12,13]	Data are insufficient [14,15]
Mechanism of action	The inactivated vaccine contains the killed “SARS-CoV-2 virus, recognized by the immune system, triggers a response, and builds immune memory” to fight SARS-CoV-2 [10,11]	The inactivated vaccine contains the killed “SARS-CoV-2 virus, recognized by the immune system, triggers a response and builds immune memory" to fight SARS-CoV-2 [12,13]	The inactivated vaccine contains the killed “SARS-CoV-2 virus, recognized by the immune system, triggers a response, and builds immune memory” to fight SARS-CoV-2 [14,15]
Indications	For active immunization against SARS-CoV-2	For active immunization against SARS-CoV-2	For active immunization against SARS-CoV-2
Contraindications	Known history of anaphylaxis, if developed after the first dose should not receive a second dose and acute symptoms [10,11]	Known history of anaphylaxis, if developed anaphylaxis after the first dose and should not receive a second dose [12,13]	Known history of anaphylaxis, if developed, should not receive a second dose, and acute infection or fever [14,15].

**Table 2:** Comparison of immunogenicity and adverse effects between the Sinopharm, CoronaVac, and Covaxin vaccines against COVID-19 infection.

Characteristics	Sinopharm	CoronaVac/Sinovac	Covaxin
Immunogenicity/neutralizing antibodies/duration of immunity	The median level of antibody and IgG level increased from 11.12 to 2607.50 and 4.07 to 619.20 BAU/mL on day 14 [16]	Neutralizing antibodies to live SARS-CoV-2; 77.9% seroconverted was by 28 days after the second dose [17]	Two doses of vaccines with an interval of 28 days showed 95.0% seropositivity to anti-spike antibodies. [18,19]
Local adverse effects	Pain at the injection site, redness, [20]	Injection site pain (41.5%) [21]	Injection site pain, swelling, redness, and itching [15]. Adverse effects were mild or moderately common after the first dose [22]
Systemic adverse effects	Fatigue, headache, myalgia, general lethargy, body ache, arthralgia, nausea, chills, fever, dizziness [20,23,24], and thromboembolism [25]	Fatigue, headache, muscle pain, and joint pain were common systemic effects [21]. In total, 57.49 per 100,000 people with thromboembolism [26]	Headache, fever, malaise, body aches, nausea, vomiting [15], myocarditis [27], and facial paralysis [28]

## Data Availability

Not applicable.
